# Relationship between nutrition and reproduction

**DOI:** 10.1002/rmb2.12332

**Published:** 2020-06-15

**Authors:** Fumitoshi Koga, Shigeki Kitagami, Arisa Izumi, Tomoko Uemura, Osamu Takayama, Tsuyoshi Koga, Toru Mizoguchi

**Affiliations:** ^1^ Koga Fumitoshi Women’s Clinic Fukuoka Japan; ^2^ Shinjyuku Mizoguchi Clinic Tokyo Japan

**Keywords:** AGEs, diet, fertility, insulin resistance, nutrition

## Abstract

**Background:**

Recently, the relationship between nutrition and reproduction is being studied. In particular, when older women receive reproductive treatment, egg aging causes greater problems than organic factors.

**Methods:**

This study investigated the relationship between nutrition and reproduction with a focus on factors that cause aging, including oxidation, glycation, and chronic inflammation. A large volume of data concerning each nutrient's relationship with reproductive medicine was collected from a number of observational studies.

**Main findings:**

The results showed that refined carbohydrates should be avoided and care should be taken to achieve proper intake of omega‐3 fatty acids. Folic acid and vitamin D were also effective. For men, antioxidant measures are especially effective. The effects of antioxidants are related to insulin resistance, which causes chronic inflammation.

**Conclusion:**

Recent research has shown that rather than meal content, meal intervals are more important for improving insulin resistance. Future research should examine lifestyle‐related nutrition factors and their relationships to reproductive treatment.

## Graphical abstract

Recent research has shown that rather than meal content, meal intervals are more important for improving insulin resistance and reproduction.

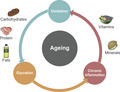

## 1 INTRODUCTION

Until recently, a low‐calorie low‐fat diet was considered an effective dietary therapy to improve obesity. However, a randomized study published in 2006 ^1^ examined this gold standard for dieting: “lower calories and fat and increase activity!” A total of 50,000 post‐menopausal women were recruited, and the results were studied extensively. The prospective observational study compared the target group, who underwent a 20% reduction in calories, increased activity, and reduced fat intake, with the control group, who continued eating a high‐fat high‐calorie diet for a 7‐year period. The results showed that although bodyweight in the target group was reduced by 1.8 kg after the 1st year on the plan, the subjects’ bodyweight began increasing slowly from 2 years after the start of the study onward; at the end of the 7‐year period, the average bodyweight of the target group of women was not different from those in the high‐calorie high‐fat meat group. In other words, this large‐scale experiment was a failure.

There is disagreement in industry and a great deal of debate regarding the dietary recommendations of the American government, which call for “limiting intake of fat, especially saturated fat, and taking in 55 to 60% of calories from carbohydrates,” but in spite of this, these diet types have been widely adopted. Even though overall fat intake has decreased, over 30% of US adults are now obese, and the McGovern Report questioned this development.^2^ In the 2017 report by Lancet, increased intake of sugar correlated with increased mortality rates.^3^ This follow‐up survey observed a total of 130,000 subjects in 18 countries from five continents over a 4‐year period, and the total intake of carbohydrates correlated with increased overall mortality rates, while fat intake correlated with decreased mortality. Saturated fatty acids were found to be inversely correlated with stroke, and a high‐sugar low‐fat diet was found to increase the risk of conditions such as heart disease and stroke. In other words, the diet we all learned and considered healthy was actually the opposite.

It is difficult for nutrition and medical research to clarify meal content, volume, and consumption timing since many subjects and long‐term observation are required. Interpretation of the results can be easily influenced by various industries. Even for research conducted over an expansive time period, there are numerous risks for misleading interpretations related to nutrition and reproduction. Thus, this review aimed to investigate the relationship between nutrition and reproduction with a focus on factors that cause aging, such as oxidation, glycation, and chronic inflammation.

## 2 METHODS

This review considered observation research conducted up until now that focused on clarifying aspects of nutrition and reproduction, looking back on findings in the literature for the individual nutrients of carbohydrates, protein, and fat, as well as vitamins such as vitamin D and folic acid. This review considered observation research conducted up until now that focused on clarifying aspects of nutrition and reproduction, looking back on findings in the literature for the individual nutrients of carbohydrates, protein, and fat, as well as vitamins such as vitamin D and folic acid, minerals, and toxic metals, listing the known effects of these nutrients on the fetus while investigating the extent of their involvement in fertility.

The study also focused on the core problem of reproductive medicine field and aging and considered the roles of oxidation, glycation, and chronic inflammation in this process. The study also focused on aging, which is the core problem in the field of reproductive medicine, and considered the roles of oxidation, glycation, and chronic inflammation in fertility, investigating existing studies on their effects on infertility, as well as the effects and associations of each type of nutrient. Especially for chronic inflammation, there are new findings related to insulin resistance in the field of internal medicine. Since obesity is highly related to reproductive medicine, this study also reviewed research related to dietary therapy to prevent increase in insulin sensitivity and considered the possibility of applying this therapy in the field of reproductive medicine.

## 3 RESULTS

### 3.1 Relationships between reproduction and specific nutrients

#### 3.1.1 Carbohydrates

Carbohydrates include monosaccharides such as glucose, fructose, and galactose; disaccharides such as sucrose and lactose; and polysaccharides such as starch and dietary fiber. In recent times, product additives contain notably large amounts of artificial sugars, causing people to inadvertently consume a large volume of carbohydrates.

##### Intake of whole grains and assisted reproductive technology (ART) results

The Environment and Reproductive Health (EARTH) Study, a prospective cohort study conducted by Harvard University, found that a higher intake of whole grains correlated with increased implantation and live birth success rates. This is speculated to be because a higher intake of whole grains produces a thicker endometrium, which improves receptivity, in turn increasing implantation success rates.^4^ The mechanism could be caused by the abundant micronutrients or dietary estrogen content of whole grains, by the abundant dietary fiber improving glucose metabolism or insulin resistance, or by other factors. At present, the mechanism is unclear.

##### Sugars

Moreover, since refined sugars can easily elevate blood glucose, ART success rates are lower among women who regularly consumed foods high in refined sugars.^5^ In addition, other findings suggest that the intake of drinks that contain artificial sweeteners has a negative effect on ICSI results.^6^

#### 3.1.2 Fats

Fats are an important nutrient that, along with carbohydrates, provides energy for activities of daily living. Fat cells serve as energy storage. Fats constitute cell membranes and play a role in stabilizing the functions and physiological actions of the body. The main components of fats are fatty acids, which are divided into two main categories, saturated and unsaturated fatty acids, based on structural differences. Animal foods contain large amounts of saturated fatty acids, and unsaturated fatty acids are mostly contained in plant‐based oils. Unsaturated fatty acids include omega‐3 fatty acids such as DHA, EPA, and α‐linolenic acid; omega‐6 fatty acids such as linoleic acid and γ‐linolenic acid; and omega‐9 fatty acids such as oleic acid.

##### Omega‐3 fatty acids and ART results

Since 1950, animal fat intake has decreased, and intake of vegetable oils has increased since these products are flooding the market at low prices. Within polyunsaturated fatty acids, omega‐3 and omega‐6 fatty acids are completely different substances, and there have been findings related to the differing roles each type plays in the body.^7^ Studies since around 2011 reported that intake of omega‐3 fatty acids improves embryo quality and implantation rates in both humans and animals.^8^, ^9^, ^10^ However, in Western observational studies conducted in the United States and Denmark, volumes of omega‐3 fatty acid intake differed, and there were questions about the actual clinical data.^11^ Although there have been questions about the accuracy of information about fatty acid intake compiled from questionnaires, in the 2018 EARTH Study, gas chromatography was used to measure blood concentration on days 3 and 9 during induced ovulation, and results of fatty acid analysis showed that patients who consumed food rich in docosahexaenoic acid (DHA) and eicosapentaenoic acid (EPA) omega‐3 fatty acids and had high blood concentration were more likely to have successful ART outcomes, and EPA was reported to be linked to high live birth rates when its concentration was high.^12^ In addition, in pregnant mothers who took DHA supplements, their children have reduced incidence of attention‐deficit/hyperactivity disorder ^13^ and enhanced growth and development.^14^

#### 3.1.3 Protein

Proteins are composed of approximately 20 different amino acids and are essential to the body. As the enzymes that digest proteins are derived from proteins, people who have protein deficiency may have difficulties digesting and absorbing proteins.

##### Fertility and intake of protein‐rich food

Although consuming sufficient protein is widely considered important in recent years, few studies focused on protein type, intake volume, and its relationship with reproduction. In reports based on questionnaire data, women who consumed large amounts of vegetables and fish had high rates of blastocyst formation; on the contrary, red meat, alcohol, and smoking correlated with lower rates. In addition, aggressive dieting was linked to lower rates of blastocyst formation.^15^ The Mediterranean diet is considered a healthy way of eating; it is composed mainly of fish and poultry, with limited red meat and dairy products. This diet also contains large amounts of whole grains and olive oil and has been reported to improve ART success rates.^16^ Among non‐obese women under 35 years, the live birth rate among those following a Mediterranean diet was 20% higher than among those using other dietary approaches. However, no significant difference was found in women aged > 35 years, and for all age groups, no differences were seen in egg collection or embryo grades.

Another EARTH Study focused on how protein type and quantity are related to the number of follicles.^17^ This study reported that infertile women with higher intake of dairy‐derived proteins tended to have fewer antral follicles. This study also speculated that steroid hormones contained in milk could be the problem. It is currently unknown whether the effects of casein‐induced increase in intestinal permeability could be a factor.^18^ In animal experiments, excessive intake of galactose induces premature ovarian failure.^19^ However, intake of dairy products has been reported to increase live birth rates,^20^ but this is still a disputed area.

#### 3.1.4 Vitamins

##### Vitamin D

Vitamin D is generated within the body when 7‐dehydrocholesterol, which is synthesized from cholesterol, is exposed to ultraviolet (UV) light, causing the formation of vitamin D_3_ precursors in the skin. After the heat exposure, these precursors generate activated vitamin D_3_.

Vitamin D deficiency causes a decline in the absorption of calcium and phosphorous from the digestive tract, leading to decreased reabsorption of calcium by the kidneys, which causes reduced calcium concentration in the blood. As this can cause rickets in children, the importance of vitamin D during pregnancy is well known. In recent years, vitamin D receptors have not only been detected in the bones, but also in other parts of the body, suggesting that vitamin D could be involved in the prevention of diseases such as breast cancer, colon cancer, diabetes, allergy to pollen and other materials, and autoimmune disorders.

Vitamin D deficiency correlated with increased risk for abnormal pregnancy and increased risk of obstetric complications such as preeclampsia and fetal growth restriction.^21^ Vitamin D is also thought to have a protective effect for premature births.^22^, ^23^ In addition, intake of vitamin D during pregnancy has been reported to increase birth weight and height of the newborn.^24^ For patients undergoing ART, vitamin D deficiency has been reported to have negative effects on egg and embryo quality, as well as reproductive function.^25^, ^26^, ^27^ When anti‐Müllerian hormone (AMH) and vitamin D were measured in premenopausal women, a positive correlation was found between AMH and vitamin D concentration among those aged ≥ 40 years, and the data have shown a correlation between vitamin D deficiency and decreased AMH.^28^ Vitamin D is also thought to play an important role in endometrial receptivity and implantation.^29^ The rate of vitamin D deficiency was also significantly higher among women experiencing recurrent miscarriages, which increased the risk of abnormalities in auto‐immunity and cellular immunity.^30^

On the contrary, vitamin D has been reported to have no effect on pregnancy rates following transfer of thawed frozen embryos,^31^ and in infertile women, blood concentration levels of vitamin D did not correlate with AMH, which is a marker of ovarian reserve, and the number of antral follicles.^32^ When implementing euploid embryo transfers, no correlation was found between vitamin D levels and pregnancy success rates, and there have been other contradictory reports.^33^ In the UK, Chu et al conducted a meta‐analysis related to whether vitamin D concentration affected ART live birth rates.^27^ Eleven cohort studies were analyzed, five of which were prospective and six were retrospective, and the total number of patients in all studies was 2,700. The level of 25(OH) vitamin D of the subjects was categorized as deficient at 20 ng/ml (34.6% of subjects), insufficient at 20‐30 ng/ml (45.3% of subjects), and sufficient at > 30 ng/ml (25.7% of subjects). In addition, the live birth rate was significantly higher in the vitamin D sufficient group than in both insufficient group and deficient group. No significant difference in miscarriage rates was identified.

##### Folic acid

Folic acid assists cell production and regeneration through its involvement in the synthesis of proteins and nucleic acids. In addition, folic acid and vitamin B_12_ assist the generation of red blood cells. In folic acid deficiency, red blood cells cannot be produced normally, and this can cause anemia, immune system functional decline, and vascular diseases.

Since the 1990s, a daily intake of 400‐800 μg of folic acid has been recommended for women of childbearing age to prevent neural tube defects,^34^ and the effects of folic acid on neural tube defects have also been reported.^35^ Folic acid is involved in deoxyribonucleic acid (DNA) production and plays a role in processes such as gamete formation, fertilization, and pregnancy.^36^, ^37^ Although intake of folic acid within a specific time period has been reported to increase rates of spontaneous miscarriage, further research suggested that it actually reduces spontaneous miscarriage rates.^38^, ^39^, ^40^

As regards the effectiveness of folic acid intake in women undergoing ART, patient groups with daily intake of ≥ 800 μg have a 20% higher rate of live births than groups with daily intake of ≤ 400 μg.^41^

Folic acid deficiency causes megaloblastic anemia when occurring in conjunction with vitamin B12 deficiency, and sufficient intake of vitamin B12 and folic acid has been shown to protect against the negative effects of environmental hormones.^42^

#### 3.1.5 Minerals

##### Zinc

There are more than 3,000 enzymes in the human body. Zinc is deeply connected with more than 300 enzymes that are involved in various body processes, including cell division and metabolism, as well as the maintenance of healthy skin and hair, taste, immunity, and sexual function. Zinc is also essential for the metabolism of sugars and the breakdown of alcohol, and the amount of zinc required for these processes is dependent on the individual's diet. Although serum zinc measurement is generally used to ascertain blood zinc content, because blood cells contain large amounts of zinc, rapid serum separation is necessary to avoid the elution of zinc from within these cells into the serum.^43^ Moreover, since the activity of zinc enzymes is decreased in zinc deficiency, alkaline phosphatase is also used for serum zinc evaluation.^44^ Zinc deficiency may increase the risk of asthenospermia in men.^45^ However, there are reports that zinc administration may increase sperm count in these cases.^46^ By contrast, other reports stated that the administration of supplemental zinc and folic acid had no effect on sperm findings.^47^ Unlike vitamins, minerals are affected by digestive function and the buildup of toxic metals. Because this indicates that absorption rates differ from person to person, results tend to be statistically different.^48^

Zinc is considered essential for fetal development; it has been reported that the serum zinc levels of the mother tend to decline in the course of pregnancy.^49^ Because zinc concentration is high in the umbilical cord blood, its supply in pregnant women is presumed to prioritize the fetus. In addition, supplemental zinc administration has been shown to reduce the incidence of premature births.^50^ Although there are few reports of increased pregnancy rates in infertile women supplemented with zinc, a study from Australia investigated blood concentrations of zinc, selenium, and copper at week 15 of pregnancy and compared the results with the length of infertility.^51^ According to that study, while women with low levels of selenium and zinc tended to have longer periods of infertility, there was no association between infertility and copper concentration. Selenium is considered to have an antioxidant function, along with vitamin E.

Regarding ICSI, intracellular calcium and extracellular zinc concentrations increase simultaneously when the egg cell becomes active.^52^ This phenomenon is known as the “zinc spark” and has also been observed in egg cells of mice. Zinc spark is considered to have an impact on the quality of the fertilized egg, suggesting that zinc is important for fertility.

#### 3.1.6 Toxic metals

The relationship between toxic metals and the recent increase in autism spectrum disorders in children has been investigated.^53^ Further, in 2011, the US government issued an advisory that pregnant women should limit their fish consumption due to the risk of mercury contamination and, subsequently, fish consumption declined.^54^ Blood concentration of mercury among women of reproductive age has been reported to have no relationship with fertility.^55^ However, toxic metals are absorbed in daily life through exposure to various materials, not just large fish. A study in Taiwan reported that infertile women had higher blood concentrations of lead and arsenic than pregnant women, and this was presumed to be the result of day‐to‐day intake of alcohol and traditional Chinese herbal medicines.^56^

In a study that investigated the methylation patterns of DNA, blood concentrations of mercury, lead, cadmium, and bisphenol A were measured on the date of egg extraction and these toxic metals were found to affect methylation, even at low concentrations.^57^ Mercury impairs the body's ability to transport and utilize essential minerals, and lead and cadmium are known to have similar effects. Additionally, a study that investigated the concentration of heavy metals and trace elements in the blood and antral fluid through the ART process reported that high blood concentrations of lead and high antral fluid concentration of copper were associated with low pregnancy rates.^58^ In addition, another report suggested that when blood tests prior to ART revealed high concentrations of lead and cadmium, it was associated with reduced pregnancy rates.^59^ Although hair mineral testing is used to assess mineral concentrations in the body and in excretion conditions, this test is not considered standard. Further research is needed to determine the relationships between heavy metals and vitamins and minerals, as well as their association with pregnancy.

### 3.2 Relationships between nutrition and reproduction from the perspectives of oxidation, glycation, and chronic inflammation

#### 3.2.1 Contribution of oxidation

Oxidative stress is defined as harmful effects on the body caused by oxidation reactions—the balance between reactive oxygen and the antioxidant system (antioxidant compounds). Consumed nutrients are broken down inside the body, and the mitochondria inside the cells convert them to energy through oxidation reactions. When excessive amounts of reactive oxygen are generated through this process, oxidative stress occurs; this process is currently attracting attention as a cause of aging. Antioxidant compounds have an antagonistic and neutralizing effect on reactive oxygen, which prevents damage to cells and DNA from oxidative stress.^60^ Reports suggest that vitamins C and E are antioxidant compounds that may affect successful pregnancy.^61^ Multivariate analysis has shown that increased intake of vitamins C and E may shorten the time required to achieve pregnancy. In women aged < 35, increased intake of vitamin C was used to attempt to shorten the time required to achieve pregnancy, and > 35 years, increased intake of vitamin E was administered to achieve early‐stage pregnancy. A Cochrane meta‐analysis reported the results of randomized controlled trials on the effects of these antioxidants. The results found no evidence of increased live birth rates when subjects administered antioxidants were compared with those receiving a placebo.^62^ At present, there is no sufficient evidence to support the routine use of antioxidants in patients undergoing in vitro fertilization (IVF). However, if reactive oxygen increases, the lifestyle habits and stress that caused such an increase are not improved, and for this reason, designing a research model to determine whether antioxidants administered at normal volumes contribute to pregnancy is a difficult prospect. Smits et al also report that oxidative stress and the pathophysiology of low fertility are related and antioxidants could possibly reduce the damage caused by oxidative stress, but they provided no high‐quality evidence to indicate the advantages and disadvantages.^63^

For IVF culture, Garder et al ^64^ reported that the use of antioxidants increased the rate of embryo growth from the 2‐cell stage to the diastolic embryo stage; as a result, the number of cells in the blastocyst was increased, and the level of hydrogen peroxide in the cells was decreased. In this mouse experiment, when the antioxidants acetyl‐l‐carnitine, n‐acetyl‐l‐cystine, and α‐lipoic acid were introduced to egg processing and cell culture, increased embryo development and blastocyst cell numbers demonstrated a direct effect on the eggs. In addition, the combined use of antioxidants has been reported to maintain intracellular glutathione levels up to the 5‐cell development stage, increasing the rate at which eggs develop into blastocysts.^65^ Recently, a culture solution that contains antioxidants is being applied in clinical settings.

#### 3.2.2 Antioxidant initiatives for the male partner

Regarding semen findings, analyses showed that reactive oxygen levels were increased and antioxidant levels were decreased among infertile men compared with fertile men.^66^, ^67^ In addition, adding the antioxidant resveratrol when freezing semen is a possible means of avoiding oxidative damage for frozen human semen.^68^ Thus, promoting the intake of foods that contain omega‐3 fatty acids, sufficient antioxidants, and nutrients such as folic acid, vitamin B12, and zinc that are involved in carbon metabolism is important for improving male fertility.^69^ In addition, appropriate exercise and sufficient intake of food containing omega‐3 fatty acids, antioxidants, folic acid, calcium, and vitamins promote the release of nitric oxide, which has been reported to be effective for improving conditions such as erectile dysfunction and vascular disorders.^70^ Moreover, if the antioxidant vitamin E is combined with the anti‐estrogen drug clomiphene citrate and administered to patients with idiopathic poor‐quality sperm or asthenospermia, pregnancy success rates significantly increased and both total sperm count and forward‐moving sperm count improved.^71^

#### 3.2.3 Contribution of glycation

As a mechanism for the onset of diseases such as obesity, insulin sensitivity, and diabetes, advanced glycation end products (AGEs) are getting attention. These compounds also cause aging.^72^ AGEs are substances produced within the body when excessive sugars consumed in meals are combined with proteins.^73^ The creation of AGEs within the body is an irreversible process based on blood glucose control and continuity, and because they are only metabolized extremely slowly, their accumulation is related to “records of high blood glucose.” Furthermore, AGEs themselves enhance the expression of receptors for AGEs (RAGE).^74^

Eggs are preserved from the fetal stage and affected by aging caused by accumulation of AGEs.^75^ Higher levels of AGEs in the blood and follicular fluid have been reported to cause slow follicle formation, lower fertilization rates, and worsen embryo development, resulting in deceased pregnancy rates. Soluble RAGE in the follicular fluid has also been shown to be positively correlated with follicular fluid AMH levels. High‐soluble RAGE leads to a collection of a larger numbers of eggs, resulting in higher pregnancy rates.^76^ Association with infertility has also been pointed out.^77^ Vascular endothelial cells have AGE receptors; thus, they are easily damaged by glycation. Glycation cannot be identified by conventional glycated hemoglobin and fasting blood glucose, but since these compounds are easily produced when blood glucose rises, it is thought to be highly correlated with diet.

#### 3.2.4 Contribution of chronic inflammation

Recently, the involvement of chronic endometritis in recurring implantation disorders is getting a lot of attention.^78^ In addition, antibiotics have been suggested to be an effective means for treating chronic endometritis.^79^, ^80^ Until now, the endometrium was thought to be a sterile environment, but Moreno et al showed that it contains specific microbiota. Among the patients achieving successful live births through IVF, lactobacillus species comprised the majority of the endometrial flora, while other types of bacteria proliferated in the unsuccessful patient group.^81^ This irregularity in intrauterine flora was found correlated with reduced success rates among patients with recurring implantation dysfunction, and its relationship with chronic endometritis is currently a research focus.^82^ The treatment of this intrauterine flora irregularity could contribute to improving pregnancy rates.^83^

Although chronic endometritis is getting attention in the field of reproductive medicine lately, the role of inflammation in various chronic diseases such as ulcerative colitis, Crohn's disease, and inflammatory bowel diseases, as well as in arteriosclerosis, depression, dementia, and atopic dermatitis, has been investigated.^84^ Inflammation occurs when tissues are damaged by reactive oxygen, and oxidation and inflammation are deeply interconnected. When AGEs are formed through glycation, they express reactive oxygen, causing inflammation. Oxidation, glycation, and inflammation thus accelerate aging synergistically. In addition, this study considered insulin resistance as a factor related to inflammation.

Obesity rates are increasing, so does the rate of obesity among infertility patients.^85^ Increased body mass index reduces sperm concentration and total motile sperm count ^86^, ^87^ and has adverse effects on semen quality.^88^ For women, insulin sensitivity that coincides with obesity inhibits ovulation and reduces the quality of eggs and embryos,^89^, ^90^ which is thought to have adverse effects on implantation.^91^ IVF live birth rates have also been lower among obese couples.^92^ The bodyweight of couples is correlated, with many couples exhibiting the same degree of obesity due to similar diet and exercise patterns. Since targeting only the female partner frequently fails, providing guidance for both partners has been suggested as a better approach.^93^ However, many weight loss programs have no clear benefits in terms of IVF success rates.^94^ Thus, a randomized study comparing the expenses and effectiveness for achieving a healthy live birth within 24 months among patients showed that changing lifestyles before beginning fertility treatments was not superior to immediately starting treatment.^95^ However, there is still no established solution concerning the proper volume of food and frequency of meals for losing weight.

As a background factor to the global low‐carbohydrate diet trend, the DIRECT Trial in 2008, a randomized controlled trial, verified the results of dietary intervention on body fat and weight loss, as well as metabolic effects. The dietary interventions tested were the low‐carbohydrate, Mediterranean, and low‐fat diets.^96^ The results showed that low carbohydrate diet had effects on the same level as the Mediterranean diet. This showed that carbohydrates raise blood glucose levels, and protein was determined to be irrelevant. However, insulin is related to increased bodyweight and obesity. Recently, as low‐carbohydrate diets have become increasingly popular, people realized that controlled blood glucose level will prevent excessive release of insulin. However, blood glucose is not the only stimulus that causes the secretion of insulin. In 1997, in addition to insulin secretion factors, protein also causes insulin secretion, and this is completely unrelated to the glycemic index.^97^ Food that passes through the digestive tract is known to cause secretion of incretin, which in turn causes an increase in insulin secretion.

Sugar is the typical food considered to promote increased insulin resistance. Excessive intake causes diabetes,^98^ and this is true for sweetened beverages.^99^ Simply stopping children from drinking sweetened carbonated beverages has reduced obesity rates in numerous observational studies.^100^, ^101^, ^102^ The fructose contained in sugar and artificial sweeteners is a major contributor to insulin resistance.^103^ On the contrary, dietary fiber, which inhibits the release of glucose from the liver and suppresses insulin, should be consumed.^104^ Dietary fiber also increases the volume and calories discharged by the large intestine.^105^ In other words, carbohydrates are not harmful, but it is the loss of dietary fiber caused by processing and freezing that causes them to rapidly increase blood glucose and cause insulin resistance.^106^, ^107^ Taking two teaspoons of vinegar before a meal containing carbohydrates has been reported to reduce insulin by 34%.^108^

Although the glycemic index of dairy products is low, they cause insulin release in extremely large quantities. Milk contains two types of milk proteins, whey and casein, and whey protein causes increased secretion of insulin by elevating glucagon‐like peptide 1.^109^, ^110^ In this way, whey protein, meat, and fish promote secretion of large amounts of insulin even though these foods do not elevate blood glucose. A large‐scale survey found that regardless of whether it was processed or not, intake of excess red meat daily causes increases in bodyweight.^111^, ^112^ With regard to fats, no correlation was found between obesity and high‐fat dairy products,^113^ and other foods eaten with fats have reported to reduce the rise in blood glucose, preventing excessive secretion of insulin.^114^ Focusing on the details of what kind of meals increases insulin increase secretion in an approach similar to the nutrient selection choices discussed above is related to reproductive medicine.

According to Duffy et al, for insulin resistance, meal frequency is twice as important as meal content.^115^ Although refraining from consuming processed sugars and proteins is naturally important, the effects of when meals are eaten and the time interval between meals on insulin resistance improvement are currently getting attention. In other words, the focus is not only on what to eat but also when to eat. Mice experiments have shown that fasting improves glucose tolerance and reduces inflammation in adipose tissue.^116^ In humans, a short‐term high protein low carbohydrate and intermittent fasting have been suggested to produce larger amounts of weight loss.^117^ In addition, after insulin resistance becomes severe in diabetes, excess glucagon is known to cause difficulty in controlling blood glucose ^118^; the relationship of these changes to reproduction is a topic for future study.

Table 1 summarizes the current knowledge on the effects of nutrients on fertility and cause.

**Table 1:** The effects on fertility are listed and compiled by nutrient and cause

Type	Item	Effect on fertility	Reference
Nutrient	Carbohydrates	Refined sugars, sugar ↓	^5^
		Drinks containing artificial sweeteners ↓	^6^
		Whole grains ↑ (effects on implantation rate)	^4^
	Fats	Omega‐3 fatty acids (EPA, DHA) ↑	8, 9, 10, 12
	Protein	Mediterranean diet (fish and poultry) ↑	15, 16
		Red meat ↓?	15, 16
		Dairy products ↓?	17, 19, 20
	Vitamins	Vitamin D ↑?	25, 26, 27, 28, 29, 31, 32, 33, 34
		Folic acid ↑	^42^
	Minerals	Zinc ↑	^52^
		(for male infertility ↑↑)	46, 47, 48
		Mercury ↓	56, 57, 58
		Lead ↓?	58, 59, 60
	Toxic metals	Arsenic ↓?	^57^
	Oxidation	Vitamin C ↑?	42, 62, 63
		Vitamin E ↑?	42, 62, 63
		(Embryo culture ↑)	65, 66
		For male factors, omega‐3 fatty acids, vitamins C and E, and zinc ↑	67, 68, 70, 71, 72
		Refined sugars, sugar, and other carbohydrates that cause hyperglycemia ↑	76, 77
		Cooking method and eating method ↑↓	73, 74
		Related to oxidation and glycation as well.	79, 85
		Processed sugars, sugar, and sweetened drinks ↓	90, 91, 92, 99, 100
Cause	Glycation	Red meat ↓	112, 113
	Chronic inflammation	Dairy products (whey protein) ↓	110, 111
	Especially insulin resistance	Number of meals and interval between meals ↑↓	116, 117, 118

## 4 CONCLUSIONS

Intake of sugars and processed foods that can cause rapid changes in blood glucose should be avoided. Proactive intake of omega‐3 fatty acids is effective, and fish is a good food source for protein. In order to ensure proper absorption of essential minerals such as zinc, digestive health must be maintained. Elimination of toxic metals that inhibit absorption may also be effective, but further research is needed to verify this. Based on insulin resistance research, considering the insulin secretion response to food choices rather than simply thinking about the increase and decrease in blood glucose is likely to be beneficial for reproductive medicine. The time between meals and insufficient snacking has been determined to be more important than meal content, and insulin resistance and related reproductive results are important topics for future research. The accumulation of heavy metals, periodontal disease, inflammation due to nasal pharyngitis or *Helicobacter pylori*, and obesity are all known causes of chronic inflammation. In addition, the effects of intestinal candidiasis cannot be overlooked. Especially since the digestive tract is responsible for 70% of immune functions, aiming for a diet that achieves a favorable balance of intestinal flora is likely to be a solution. Establishing optimized nutrition for each of these individual causes is necessary.

## DISCLOSURES

*Conflicts of interest:* Fumitoshi Koga, Shigeki Kitagami, Arisa Izumi, Tomoko Uemura, Osamu Takayama, Tsuyoshi Koga, and Toru Mizoguchi declare that they have no conflict of interest. *Human rights statements and informed consent:* All procedures followed were in accordance with the ethical standards of the responsible committee on human experimentation (institutional and national) and with the Helsinki Declaration of 1975, as revised in 2005. Informed consent was obtained from all patients for being included in the study. *Human/animal studies:* This article does not contain any studies with human or animal subjects performed by any of the authors.
